# The Phenomenon of Loneliness in Old Age

**DOI:** 10.11621/pir.2021.0310

**Published:** 2021-09-30

**Authors:** Valentina A. Naumova, Zhanna M. Glozman

**Affiliations:** a Vitus Bering Kamchatka State University, Petropavlovsk-Kamchatsky, Russia; b Faculty of psychology, Lomonosov Moscow State University, Moscow, Russia

**Keywords:** General loneliness, positive loneliness, solitude, gerontological cohort, loneliness as a resource for self-cognition and self-development

## Abstract

**Background:**

The issue of solitude is fragmentary in gerontological investigations, and is generally interpreted as loneliness: a negative experience of lack of relationships with other people.

Ageing people have many variants of loneliness, often connected with their own prejudices or satisfaction with their social contacts.

In loneliness, opportunities and rights to the sovereignty of one’s life space can be preserved.

**Objective:**

To study loneliness as a fact of life, a multi-dimensional phenomenon, including the feeling of loneliness itself, lack of communication, and ability to be alone. We suppose that senior adults with different levels of psychological well-being are specific in this acceptance of loneliness and ability to find resources in this situation.

**Design:**

The participants comprised 129 residents of Kamchatka Region aged 60–82. In the first stage, using C. Ryff’s “Psychological Well-Being Scale” with mid-values cluster analysis, the respondents were divided into groups with different levels of psychological well-being.

In the second stage, the data of the “Differential Questionnaire on Experiencing Loneliness” and “Subjective Perception of One’s Own Life” questionnaire were used for correlation analysis of interrelations between psychological well-being and the “positive loneliness” subscale, revealing the participants’ ability to find resources in loneliness.

**Results:**

The research shows that experiencing loneliness in the gerontological cohort is non-homogeneous; it is interconnected with personal attitudes towards positive loneliness, with psychological well-being. It changes the activities of the elderly and the extent of experiencing loneliness.

**Conclusion:**

There is cultural mitigation of loneliness in gerontological cohorts and also in their shift from a negative mindset towards an existential one.

## Introduction

Rapid growth of the proportion of the world’s ageing population is becoming more and more obvious, which is why the first decades of the 21st century have been a time of increasing research interest in problems of ageing and senior adulthood. The previously dominant bio-determining model of old age as an illness and involution are now giving place to an adaptive-compensative model, considering old age to be a stage in human evolution and underlining that the elderly have a wide range of potential resource mechanisms for compensation for negative consequences of ageing and for continuing development of the person. Works by R.J. Havighurst (1961) and later by J.W. Rowe & R.L. Kahn (1987) were very important for the creation of this model; they demonstrated that deteriorating health cannot predict a decline in the ageing person’s subjective state. The authors reject the idea that old age is a period of illnesses and deterioration, and oppose to this the concept that old age opens perspectives for new activities. The ageing person is responsible for their own state: the more his physical and social activity, the better is his health and emotional state. The authors also propose a new understanding of age-related changes through the challenges theory, which supposes that risks of the appearance of different illnesses don’t depend only on age. The works by J.W. Rowe & R.L. Kahn created a new gerontology, looking for personal resources and potentials in the elderly.

A study by P. Baltes & M. Baltes (1990) proposed the model known as Selection—Optimization—Compensation (SOC)—three mechanisms of adaptation for the elderly. Selection means concentration on the most important tasks; optimization looks for the best ways to achieve these tasks; and compensation means replacement of what is not possible by alternative means. A combination of these mechanisms provides preservation of activity, adaptation to changes, and slows destructive and degenerative processes. The model accentuates the plasticity of the human mind during gerontogenesis and the preservation of potentials for reconstructing internal and external resources for efficient functioning.

A typical feature of the period of gerontogenesis is restriction of social networks (death of peers, professional limitation, selectivity in relationships), which makes it even more important for the ageing person to form new strategies for relationships, based on qualitative analysis of the situation—the socio-emotional theory of selectivity by L. Carstensen & D. Isaakovitz (1999). According to this theory, psychological and emotional comfort becomes the leading motivation for communication in late ontogenesis, resulting in adaptation of one’s own communications so as to minimize negative emotional experiences and to increase positive ones. The theory also looks at the person’s social networks: how to select a communicator, to optimize the time of communication, and compensative emotional preferences of communities.

Russian studies belonging to the adaptive-compensatory paradigm are represented in the adaptive-regulatory theory of V. Frolkis (1970, 1988) and by the scientific theory of gerontogenesis (Alexandrova, 1974; Ananyev, 1977, 2010; Davydovsky, 1967; Krasnova, 2011, and others).

V.V. Frolkis (1988) proved that ageing has internal contradictions: functional disturbances are accompanied by mobilization of important adaptive mechanisms, favoring stabilization of vitality, increasing activity and life expectancy.

The scientific theory of gerontogenesis underlines the social determination of ageing: the character, dynamics, and speed of age-related modifications are “realized in definite social-cultural conditions” (Krasnova, 2011, p. 116). A synthesis of self-determination and external determination is a means to influence the ageing process (Davydovsky, 1967). This is confirmed by B.G. Ananyev’s description of “the extraordinary phenomenon of ageing slowness due to social determination of human organic development in his individual life” (Ananyev, 2010, p. 137). The author pays attention to the increasing individualization of ontogenic evolution in the elderly and to “the contrast of individual types of general vital tonus and intelligence” (Ananyev, 2010, p. 229). Ananyev distinguishes two opposite orientations of ageing: convergent (destructive—causing stagnation of mental functions) and divergent (minimal loss and acquisition of new skills). This acquisition is a resource for mental intactness, for new strategies of behavior in continuation of life.

Many studies of gerontogenesis show that old age has a wide range of potential resource mechanisms for the continuing development of the person (Baltes & Baltes, 1998; Glozman & Naumova, 2014, 2018; Havighurst, 1961; Naumova, 2014; Rowe & Kahn, 1997, 2015; Settersten & Godlewski, 2016). The modern representation of the elderly includes adaptive-compensative mechanisms, together with realization of the dividends of long life expectancy as potentials and/or resources for positive ageing (Melehin, 2015; Strizhetskaya, 2016, 2018; Tornstam, 2011).

This evidence is supported by numerous variants of highly productive and successful ageing or active interaction of senior adults with the surrounding world, which can be based on positive aspects of their functioning (Kudrina, 2015; Sergiyenko & Kharlamova, 2018; Stryzhetskaya, 2016, 2018; Zavialova & Soldatova, 2019).

However, the majority of Russians still maintain that “old age has no advantages over other ages” and its image is more often stereotypically associated with inevitable losses, such that “a place of honor” and the status of being a person of great practical significance are replaced by the problem of “senior loneliness”, or such losses are viewed as an inevitable consequence of ageing, or as a widespread and unavoidable attribute of old age (Krasnova, 2005). We can probably explain this by social and economic instability in Russia and the stigmatization of the elderly as helpless, dependent, and rigid, which interferes with their social integration (Reznichenko, 2017); their financial problems, anxiety about their health, lack of institutional care and environmental adjustment for older people’s needs, decreasing their motility (Elutina & Trofimova, 2017); and the predominance of occidental-type nuclear self-sufficient families, consisting of young parents and children (Ibragimova, 2007).

These contradictions underlie our interest in the study of loneliness in old age.

Our analysis of numerous works confirms that, although the phenomenon of loneliness has been studied for a long time, it remains one of the most important issues in various scientific areas, first and foremost due to a sharp increase in the weakening of social ties and of all types of social mobility (Klinenberg, 2012; Riesman & Glazer, 1961).

In academic psychology, the aspects of loneliness have been developed in the framework of the adult attachment system (Weiss, 1973); the non-correspondence between expected satisfaction to be obtained from personal interactions and the actual level of personal relationships (Peplo & Perlman, 1989; Slobodchikov, 2007); sociocultural analysis of loneliness (Pokrovskiy & Ivanchenko, 2008); age/gender aspects of the experience of loneliness (Kon, 2008; Lyubyakin & Okonechnikova, 2016**M**; alienation mechanisms and modules of personality autonomy (Leontiev, 2011); and positive aspects of loneliness (Heidegger, 2013; Lyashchenko, 2017; Osin & Leontiev, 2016).

In more general terms, loneliness is viewed as a negative experience of one’s own lack of deep relationships with other people. This experience can occur in a community in which one lacks psychological contact (alienation), or as voluntarily accepted solitude necessary for realizing “the experience of impressions and interactions with the world” (Ishanov, Osin, & Kostenko, 2018).

The overwhelming majority of investigations of solitude and loneliness performed in gerontological groups are fragmentary and differ in their contexts: as a predictor of fast ageing (Alexandrova, 2006; Ananyev, 1977, 2010), development of degenerative processes, and mortality (Gerino, Rollè, Sechi, & Brustia, 2017; Wong, Liu, Lin, Huang, Wai, & Lee, 2016); with reference to social conditions or dwelling places (Bydtayeva & Zurayeva, 2017); psychosocial maladaptation (Nikitina & Shakirova, 2016; Reznichenko, 2017); social-emotional selectivity (Carstensen & Isaacowitz, 1999; Melehin , 2015); in theories of social capital (Biggs, Carstensen, & Kogan, 2012; Olshansky, Beard, & Börsch-Supan, 2012); and in suicidal behavior (MacLeod, Musich, Hawkins, Alsgaard, & Wicker, 2016).

Summing up, we infer that solitude in old age is an ambiguous notion, and on the whole it is presented as loneliness: a negative problem. It is also important to note that authors often join the concepts of “social isolation, social disregard for aged people” and “a deliberate choice of solitude in senior adulthood” or “experiencing loneliness in senior adulthood”. The fact of the person’s separate, independent living in old age does not automatically imply an experience of negative emotions related to loneliness as an experience of non-involvement, a feeling of being unwanted, of indifference or non-acceptance by others, according to which the person’s everyday life may be constructed (Krasnova, 2005; Yelyutina & Trofimova, 2017; Yermolayeva, 2002).

A number of researchers have noticed that people who live alone do not necessarily experience loneliness. Solitude may manifest itself with either a stereotypical negative experience or an emotionally positive one; it can become a valuable resource for “internal dialogue”, for reflective, productive activity (Ishanov, Osin, & Kostenko, 2018).

Furthermore, solitude may be considered as an important condition for analysis of the “personal vocabulary”, the core of which is formed by the person’s life experience. The consummation of one’s life experience is especially urgent in the period of gerontogenesis, as, in many respects, it determines the behavior, choices, and deeds of an ageing person (Sapogova, 2019).

Thus, many senior adults are able to manage their everyday lives without external assistance and can live on their own for a long time, “maintaining his or her independence to the utmost” (Yelyutina & Trofimova, 2017). Respondents included the following in the advantages of a “solitary life”: mobilization of functioning according to the “who-if-not-me” principle; the possibility of temporary isolation in order to assess their experience “in the context of their own life more broadly than in the ‘here and now’ under the influence of strong emotions” (Yelyutina & Trofimova, 2017).

Experiencing solitude or loneliness in senior adulthood takes a diversity of forms, and this diversity is often not immediately associated by the ageing people themselves with isolation or alienation, but rather with their own prejudice, (dis)satisfaction, or cognitive evaluation of the content and quality of their social contacts.

On the whole, solitude is characterized by senior adults as a state in which opportunities and rights to the sovereignty of one’s life space and realization of one’s life ambitions are preserved, in spite of non-acceptance, neglect, alienation, lack of understanding or indifference on the part of others (Yelyutina & Trofimova, 2017).

Thus, longer life expectancy and the phenomena of the modern ageing person bring into focus the problem field of old age support, providing opportunities for well-being in the elderly (Nilsson, Bülow, & Kazemi, 2015; Smith & Hollinger-Smith, 2015). It is also important to note that modern society is beginning to shift the focus of viewing solutions to the problem of the population’s ageing towards “cultural adaptation, which includes rethinking the interrelations between material and spiritual consumption that are changing due to longevity” (Biggs & Haapala, 2016).

The survey presented above makes it obvious that solitude in senior adulthood should be investigated not only as a negative problem, but also as a fact of life, reflecting variable attitudes of a person to loneliness and solitude, which can be a positive resource for “the formation of the whole modus of psychological life of an ageing person, his or her development of a new position in life” (Shakhmatov, 2004, p. 273).

The following ideas underlay this investigation:

- In modern society, loneliness is not the prerogative of old age, but it is in the declining years that it becomes critically significant, as at this time it is less possible to conceal one’s confusion in social relations (Victor, Scambler, & Bond, 2009).
- A conscious personal choice can be made to accept and productively exploit situations of solitude as a resource for self-cognition, internal connection with other people, nature, and God; preference for openness to change and self-transcendency are characteristic of solitude (Long, Seburn, Averill, & More, 2003).
- In senior adulthood, solitude, as an existential fact in which longevity has advantages, allows the person to “experience the world as a harmony and oneself as a harmony within that harmony” (Yermolayeva, 2002, p. 149), which, in turn, facilitates the person’s satisfactory functioning and successful ageing in general.

Based on these approaches, we suggest that late-middle-aged and elderly adults with different levels of psychological well-being (PWB) will display specificity in their attitudes towards acceptance of solitude and capability to find resources in situations of solitude. To measure the level of psychological well-being, we used the approach of C.D. Ryff, considering psychological well-being as the objective possession of the necessary psychological characteristics that facilitate more successful functioning of the subject (Ryff, 1989).

## Methodologies and Subjects

The goal of the research presented in the article was to study the solitude of the ageing person as a multi-faceted phenomenon, including analysis of the degree of loneliness and capability to find resources in situations of solitude.

The following psychodiagnostic methods were used:

1. C.D. Ryff’s Psychological Well-Being Scale (adapted by Shevelenkova & Fesenko, 2005) includes six basic scales (“positive relations with others”, “autonomy”, “environmental mastery”, “personal growth”, “purpose in life”, “self-acceptance”) and three additional ones (“affect balance”, “meaningfulness of life”, “the person as an open system”). The technique analyzes the psychological well-being of respondents at a particular stage in life (Borisov, 2019; Glozman & Naumova, 2018). Cronbach’s alpha measure of internal consistency = 0.75.
2. The Differential Questionnaire on Experiencing Loneliness (Differentsialnyi oprosnik perezhivaniya odinochestva [DOPO]) (Osin & Leontiev, 2016) makes it possible to investigate individual features of solitude and loneliness and a person’s attitude towards them. The authors of the methodology consider that solitude can include not only negative aspects (loneliness), but also needs to be analyzed as an existential factor that allows a person to value and employ solitude as a significant resource for self-actualization. The questionnaire includes eight sub-scales that form three main scales measuring the General Experience of Loneliness (the EL scale) and aspects of the attitude towards it (the DC scale [Dependence on Communication] and the PSscale [Positive Solitude]). Cronbach’s alpha measure of internal consistency = 0.81.
3. The “Subjective Perception of One’s Own Life” questionnaire (Naumova, 2014) includes questions about variables that are presumed to be relevant to experiencing loneliness (examples: “I find/don’t find a common language with my children”, “I maintain warm and friendly relationships with people who have been my friends for many years”, “I am satisfied with the relationships that have been established in my family”), as well as a number of open questions specifying states and behaviors in situations of solitude (examples: “Is the feeling of loneliness familiar to you? Could you describe it?”, “What can you be engaged in when living alone?”). The results were processed with the help of qualitative content analysis.

The group of respondents consisted of 129 residents of Kamchatsky region (88 females and 41 males) aged 60–82 (the average age is 72.3). Participation was voluntary; the investigation was held at the respondents’ residences, individually with each respondent.

## Research Design

In *the first stage*, using hierarchical cluster analysis of the Ryff Psychological Well-Being sub-scales’ standardized scores, the respondents were divided into two groups: the first experimental (EG1) and the second experimental group (EG2).

The socio-demographic characteristics of the participants are presented in *Table 1*, which shows that respondents who provided information about having a family, being employed, and living in urban conditions are predominant in EG1. No considerable differences were found in the clusters according to education and gender. The average age of participants in the first experimental group is 70.1 years, in the second group 66.4 years.

**Table 1:** Social-demographic characteristics of clusters

Characteristic	EG1, % (n = 56)	EG2, % (n = 73)	Fisher criterion ϕ*_emp_*
	**Average age**		
60–74	69.6	86.3	2.29**
75–82	30.4	13.7	2.30*
	**Gender**		
Female	64.3	71.3	1.30
Male	35.7	28.7	0.84
	**Residence**		
City	82.1	53.4	3.53**
Village	17.9	46.6	3.52**
	**Marital status**		
Married	48.2	26.0	2.61**
Divorced	33. 9	24.7	1.14
Widow(er)	17.9	49.3	3.83**
	**Education level**		
Incomplete secondary education	3.6	8.2	1. 15
General secondary education	21.4	21.9	0.08
Professional secondary education	39.3	41.1	0.22
Higher education	35.71	28.76	0.84
	**Social status**		
Works at the same place	33.9	30.1	0.46
Works at another organization	28.6	6.9	3.34**
Does not work	37.5	63.0	2.89**

*p ≤ 0.05;**pfi 0.01

Analysis of the data presenting both groups’ psychological well-being shows that the values are within the range of mid-values (according to the mean values in the sample as a whole), but comparing the two samples with the help of Student’s t-criterion shows their considerable differences (*Table 2*). Thus, in EG1, significantly higher values are registered on the PWB scales 2–6 and 8–9, and lower values on scale 7 (“affect balance”).

The considerably higher values for “affect balance” (p ≤ 0.01) found in the second experimental group can probably be interpreted as a tendency of respondents to underestimate their abilities to overcome life’s difficulties and to compromise in order to keep communications open. This situation is likely to represent selective confidence and insufficient ability to maintain positive relationships with others.

**Table 2:** Psychological well-being in the sample as a whole and in the cluster groups

Scales		positive relations with others	autonomy	environmental mastery	personal growth	purpose in life	self-acceptance	affect balance	meaningfulness of life	he person as an open system
Values for whole sample^1^ (n = 129)	Mid-value	52.76	53.81	53.33	54.12	57.77	53.01	99.68	96.86	62.48
	SD	7.29	7.76	8.60	11.48	12.08	9.14	15.36	16.44	9.78
EG1 (n = 56)	Mid-value	54.19	57.41	58.02	64.5	66.02	59.52	93.27	104.38	67.42
	SD	8.51	8.19	8.74	6.13	8.99	6.50	13.53	13.9	8.75
EG2 (n = 73)	Mid-value	51.68	51.01	49.74	46.16	51.43	48.03	104.60	91.11	58.69
	SD	6.05	6.15	6.54	7.61	10.20	7.63	14.94	15.99	8.83
Student’s t-criterion^2^		1.87	4.86**	5.93**	10.8**	8.61**	9.22**	4.51**	5.03*	5.59**

*Notes:*^1^ *standards of mean values in the sample in general;* ^2^ values are obtained by comparison of EG1 and EG2. *p ≤ 0.05; **p ≤ 0.01

On the “positive relations with others” sub-scale, no significant differences between the groups were found. The results of some other studies show that maintaining a positive attitude towards others during the stages of gerontogenesis may perform an important resource role for overcoming the difficulties or losses of ageing and maintaining active functioning of the ageing person as a subject of his or her life activity (Glozman & Naumova, 2018; Zavialova ffi Soldatova, 2019). This allows us to suggest that the possibilities for participants’ satisfactory and close relationships with others are preserved.

Summing up the results of the first stage of the research on psychological well-being, we conclude that the EG1 respondents displayed readiness to feel empathy, to establish emotionally strong relationships, and to find compromises, to regulate and control their own behavior, to plan and organize their life space, to find ways of actualizing resources for their own development, to admit and accept their internal heterogeneity. For those in EG2, their psychological well-being can be described as less satisfaction with their life circumstances (in comparison with EG1), including problems with showing warmth to others; dependence on the opinion of those around them; less control over reality, interest in life, intentions, prospects for implementing their life’s ambitions; and a need to integrate aspects of their life’s experience. The summarized data of this PWB cluster group show a tendency for passivity and a lack of sense of purpose in life.

In *the second stage* of our study, on the specificity of the respondents’ experiencing of solitude, we compared the values of the two groups, analyzing the data from the following instruments: the “Differential Questionnaire on Experiencing Loneliness (Differentsialnyi oprosnik perezhivaniya odinochestva [DOPO])” and the “Subjective Perception of One’s Own Life” questionnaire. The following research tasks were performed:

- Comparative analysis of the DOPO sub-scale values in the two cluster groups to reveal particular features of their attitude towards loneliness;
- Correlational analysis of interrelations between the PWB components and the data of the DOPO “positive solitude” sub-scale, the anticipated result of which may reveal the group specificity of the participants’ ability to find resources in solitude;
- Content analysis of the participants’ descriptions of their experience that qualitatively characterize the states and behaviors of respondents in situations of solitude.

## Results

*Table 3* presents the values of the DOPO scales in the whole sample and in the two cluster groups, and shows that the mid-values of the questionnaire scales in both groups comply with the range of mid-values of the whole sample, yet a statistically relevant difference is observed.

**Table 3:** DOPO scale values in the sample as a whole and in the two cluster groups

Scales/subscales	Values of whole sample (n = 129)		EG1 (n = 56)		EG2 ≤ n = 73)		
	Mid-value	Deviation ratio	Mid-value	Standard deviation	Mid-value	Standard deviation	Student’s t-criterion^1^
General Experience of Loneliness (EL)	34.46	8.66	31.54	9.53	36.71	7.17	1.97^*^
Isolation	12.37	4.30	11.18	4.69	13.30	3.75	2.77^*^
Sense of self	10.35	3.58	9.64	3.65	10.98	3.42	2.29^*^
Alienation	11.63	3.66	10.87	3.92	12.21	3.36	2.05^*^
Dependence on Communication (DC)	31.13	6.84	26.38	5.03	34.76	5.72	4.03^**^
Dysphoria of loneliness	9.52	2.63	8.34	2.23	10.44	2.54	5.12^**^
Loneliness as a problem	10.68	3.38	9.23	3.26	11.79	3.08	4.53^**^
Need for company	10.79	3.41	8.80	1.43	12.33	3.69	7.46^**^
Positive Solitude (PS)	22.29	8.78	29.80	6.89	16.53	4.79	6.03^**^
Joy of solitude	9.94	3.67	12.25	2.93	8.17	3.19	7.52^**^
Resource of solitude	12.15	6.05	17.37	4.65	8.15	3.36	12.55^**^

Note: **^1^**The values are obtained by comparing EG1 and EG2. ^*^p ≤ 0.05; **p ≤ 0.01;

Comparative analysis of the questionnaire scales’ and subscales’ values makes it possible to describe the characteristics of the experience of loneliness and the attitude towards loneliness in the cluster groups. Thus, EG1 respondents display *EL* (general experience of loneliness) values that are lower than the average range of the sample (*Table 3*), and considerably lower *DC* (dependence on communication) values, in the context of *PS* (positive solitude) values that are significantly higher than the average. We may suppose that the members of this experimental group are likely to be tolerant of loneliness and able to accept solitude. The respondents of EG2 display a different pattern: The quite high *EL* values are combined with considerably higher *DC* values and low *PS* values. This fact allows us to characterize the respondents as representing loneliness as suffering and a negative emotional experience, as well as possibly having problems with living in solitude without finding in it resources for their lives.

To reveal possible interrelations between the aspects of positive solitude and psychological well-being, a correlational analysis of PS parameters and PWB components was implemented in the groups (*Table 4*).

**Table 4:** Correlation of DOPO subscales of positive solitude with PWB components in the cluster groups

	Scales	positive relations with others	autonomy	environmental mastery	personal growth	purpose in life	self-acceptance	affect balance	meaningfulness of life	the person as an open system
EG1	Joy solitude of	0.028	0.177	0.227	–0.094	–0.113	0.303**	0.158	0.185	0.264^*^
	Resource of solitude	0.079	0.161	0.264^*^	0.062	–0.121	0.166	0.087	0.126	0.084
EG2	Joy solitude of	–0.151	–0.187	–0.101	0.101	–0.124	–0.188	–0.034	–0.105	–0.109
	Resource of solitude	–0.216	0.161	0.222	–0.061	0.309^**^	0.173	–0.226	0.138	0.012

^*^p ≤ 0.05; ^**^p ≤ 0.01

As is evident from *Table 4*, the correlations are not numerous. Thus, in the first experimental group we can register significant positive interrelations between the values of the “joy of solitude” subscale with the values of the PWB “self-acceptance”(p ≤ 0.01) and “the person as an open system” subscales and between the “resource of solitude” subscale with the values of “environmental mastery (p ≤ 0.05). In EG2, only one significantly positive correlation is observed, between the values of the “resource of solitude” subscale and the “purpose in life” subscale (p≤ 0.01).

Going forward, for a more comprehensive study of the respondents’ attitudes towards positive solitude, we used the questionnaire. On the basis of the respondents’ answers, their conditions and behaviors in situations of solitude were grouped into eight categories by using content analysis (*Table 5*).

The frequency analysis of the answers and the content analysis of the descriptions, which focus on the respondents’ behavior in a situation of solitude, show the

**Table 5:** Distribution of categories characterizing conditions and behaviors of respondents in situations of solitude

Categories	EG1		EG2		Fisher criterion φ_emp_
	**Examples** ^1^	(%) No.	**Examples** ^1^	No. (%)	
Health	“I go in for yoga”; “walk a lot”; “was gathering wild herbs all summer, then I will make decoctions”; “I like to take a steam-bath in the Russian sauna and think about life”; “take care of my body”; “ride a bicycle”; “take aromatic baths and give myself different facials for women’s health, it is important in our ‘peach’[*mature*] age”; “lie on pins. Did you know there is such an applicator [*Kuznetsov Acupressure Acupuncture Massage Mat*]? And what’s more, I do exercises to relieve my backache, using a stick, I have a special one”.	50 (91.1)	“I take medicine”; “constantly measure my blood pressure and sometimes blood sugar level”; “try to have more rest and eat healthy food”; “try not to think about bad things and about the future; I don’t have much time left …”; “handle my finances in order to pay the bills and get enough money for food and medicines. How else can I preserve my health?”; “keep a diary of my everyday state, write down my blood pressure and what medicines I have taken and at what time I took them. If I have doubts, I write down questions to ask my doctor”.	70 (95.8)	1.30
Tranquility	“I like to reflect”; “I like to relax; now I can do it with a clear conscience—I’ve raised them all [*my children*], hurray!”; “fishing, by all means, … stillness, tranquility, and the full pleasure of enjoying nature and life…”;“enjoy reading; I can say that I abandon myself to reading and feel so good”; “well, I feel fine being by myself, I adore such moments”.	29 (51.2)	“I sleep a lot”; “cook my favor-ite dishes … and eat as much as I want and whenever I want”; “well, I do nothing, just rest”; “I am idling my time away”; “hardly anyone is interested in us now, so I just sit and do nothing, that’s all, so the days pass”; “I’ve deserved rest, I worked a lot in my life and worked hard. Now let them take care of me and give me peace and tranquility”.	65 (89)	3.91**
Independence	“I don’t have to stay in tune with someone else’s mood”; “I feel that I am the master of my life”: “no conflicts about trifles”; “I am not burdened with care about some-one else”; “I can take a break at any time, I don’t exclude the possibility of relaxed solitude”; “so as not to be a burden, I rely only on myself”; “financial independence; cur rently I have enough funds …” “don’t need to submit to anyone”.	50 (89.3)	“I can be myself, that is‘ a former me’. I oft en return to my past; it is my past that was real…”; “solitude is when I don’t have to prove the authenticity of my memories about my own life to anybody. Well, indeed, can anyone believe that all that happened to me? As my grandson says: ‘Grandma, is it possible to believe in your fancy stuff?’”; “how bad it is to live alone; my wife died and it is not easy to live with the children; they are really different now, and they may be waiting for my death …, I don’t want to be the cause of their sinful thoughts”.	55 (75.3)	1.9*
Acknowledgement	“I want to be in tune with the times…, therefore I surf a lot on the Internet, get very much that is new and useful from it”; “accept my age with curiosity, try to listen to my body, thoughts, wishes …, this is a new and very interesting experience for me (for now, at least)”; “play computer games”.	35 (62.5)	“I look through my old albums again and again”; “I still do the home canning myself, my granddaughter praises me for it”; “I go down the stairs on my own from the fourth floor and do the shopping all myself”; “I am still in good condition to drive my car … my grand son, of course, laughs at me, but I hope, it is not malicious laughter”;”this is the cost of missed opportunities”.	22 (30.1)	2.44**
Creativity	“Now I have enough time at last, so I am making a creative revision of my many years of notes about travelling by sea. I went to sea for almost 35 years”; “I play the guitar”; “I am concerned with making a genealogical tree; I want to leave the heritage for my grandchildren”; “I bake fish pies; my friends say my pie is a masterpiece…”	48 (85.7)	“I watch TV serials”, “sometimes I sing for myself”; “repair warm socks for my husband; our grandchildren don’t wear such socks anymore …, and I can save some money from my pension”; “we listen to music”.	47 (64.3)	2.47**
Relations with the family	“I think that it is very important in our age; moreover, it is important for the whole family that my old man and I should be in good shape”; “prepare presents for everyone; now I am indeed not so lively as before”; “learn poems by heart; but now I am becoming forgetful, so I mustn’t do it; I don’t want to frighten my home-folks”; “I pray for the health of my relatives and close friends”; “I am always thinking about my loved ones, how they are, all my darlings. I am eager to help them while I can, and then—at least not to disturb them”.	39 (69.6)	“I do a good many of our house-hold routines; my home-folks work a lot”; “I think a lot and worry about my children and grandchildren …, what if I fall ill and cannot move? They will have a lot of trouble with me “; “oft en communicate with my children via Skype; they have moved far away from me, so I always keep listening, not to miss a call”.	69 (94.5)	3.4**
Education	“I attend the Third Age Institute”; “master computer graphics, gradually”; “study Chinese”; “I have been dreaming about a journey to Laos for a long time. I am planning the route by myself, so I am studying the history and geography of the country”; “I want to give myself a present for my 70th birthday–to jump with a parachute, so I am studying the material gradually”.	41 (73.4)	“I read sometimes”; “try to do crosswords, but get bored soon”; “study new recipes”; “not so long ago I learned how to make butter”.	20 (27.4)	3.27**
Terminality of life	“I recall my friends and relatives who are gone. Sometimes I think over how it can happen to me”; “I think over how to write my will correctly; I don’t want to offend anyone, and besides, I want to be remembered well”; “I often think about the final days of my life. Try to keep myself in good shape”; “A year ago my friend died. Her children quarreled over her inheritance. Nobody even wants to give money for the tombstone. I oft en think about how awful it is, get nervous, cry, but do it so that my children shouldn’t see it”.	40 (71.2)	“What joy can be found in solitude? At our age being alone is terrible and dangerous; the end of life is coming soon”; “I live alone. Sometimes it happens that nobody has been wondering how I am for several days. I think sometimes that I could die and nobody would care”; “when I was strong and earned a lot, everybody needed me. And now that I am on the edge of life, they are probably waiting for my demise”.	56 (75.3)	0.22

*Note:* ^1^ These examples of respondents’ answers are presented in the authors’ wording. *p ≤ 0.05; **p≤ 0.01

group specificity. Thus, in the first experimental group, the respondents describe in a differentiated way their experience as a readiness to find in solitude a resource for self-cognition and self-development. Being alone, the respondents of the first group display their negative feelings no more rarely than the respondents of the second group, but positive feelings are considerably more frequent. This suggests that, in general, they do not typically evaluate solitude in a negative way. Besides, uniting the few correlations of *PS* with the results of content analysis of the descriptions of solitude, we can detect the ability of the participants to use the situation of solitude as an opportunity “to be, not to seem” real and true, to experience positive emotions and accept oneself as one is.

In the second experimental group, however, the content-related analysis of the results can be interpreted as an expression of the deficiency (scarcity) of this group’s contacts with others, a critical shortage of emotionally close, intimate, and constructive relations. Their low estimation of the purposes of their own life, dependence on others, and orientation to the other people’s opinions, difficulties with fulfilling their life’s ambitions, unwillingness/inability to integrate their life experience may facilitate or even stimulate the boredom, yearning, and sadness, and, in general, actualize bitterly painful ideas about their own lonely feebleness and uselessness. The significantly positive correlation between “resource of solitude” and “purpose in life” also presents some difficulties for theoretical understanding. To provide explanations, we find it relevant to use the results of certain investigations, the analysis of which sheds light on the voluntary choice of “negative retirement solitude” (Dickens, Richards, Greaves, & Campbell, 2011; Kudrina, 2015; Miklyaeva, 2018).

We have shown that at the stages of gerontogenesis, the scarcity and indefiniteness of goals and plans, a decline in the feeling of purposefulness, a negative and non-differentiated expectation of the future are, as a rule, associated with an inescapable and unfavorable health prognosis, which may block life prospects and activate the mechanism of personality stagnation. Reality, as it has developed, makes ageing persons feel lonely even if they live with their family, are employed, whether or not they have financial difficulties, and whether they are in good or bad health. So, EG2 respondents represent themselves as lonely, describe their state either as “escape from the everyday world to memories”, or as taking umbrage at the “injustice of life”, or as a “forfeit for failures in the implementation of biographical projects and one’s own underperformance” (Yelyutina & Trofimova, 2017, p. 44). On the whole, the respondents display the experience of loneliness combined with self-restrictions and uncertainty, an inability or unwillingness to find in solitude a resource, a productive way of fulfilling one’s own opportunities and life ambitions.

## Discussion

In the existential tradition, loneliness is considered the most important challenge, which can be addressed by acceptance of solitude as a fact of life and adaptation to it, or by experiencing distress and regression of the personality. In the modern world, everyone faces quite frequent chances to experience loneliness, and loneliness is now not just a prerogative of the elderly (Klinenberg, 2012).

At the same time, the current scientific discussion of gerontogenesis reveals the emergence of positions concerning old age that express a new world-view, where the issues of loneliness remain pressing (Biggs & Haapala, 2016; Hagan, Manktelow, Taylor, & Mallet, 2014).

Thus, in the context of the more liberal occidental attitude to ageing, the rejection of stereotypes concerning the widespread and inescapable character of loneliness in old age and the necessity of overcoming it is accentuated (Blanchard & Anthony, 2013). Guaranteed social support for spatial and psychological conditions of self-realization of the ageing person can be an alternative to direct social relationships in the public space. Then the elderly may find themselves needed by “living in a community”, and through meaningful solitude as a justified distancing that creates a context for communication and social interaction. In Russian reality, an elderly person’s negative solitary life is often demonstrated along with the traditional preference for living in families, of intergenerational communication and protecting the social-cultural traditions of different generations.

In the context of ageing as “the triumph of active and productive old age”, solitude is also admitted as a withdrawal from social involvement and use of the creative benefit of inactivity for calm reasoning and critical analysis. Active ageing does not exclude “positive solitude” (Tornstam, 2011). Solitude and the possibility of meditation are essential elements for reaching a critical distance. Productive critical inactivity may provide a symbolic space for investigating semantic structures of ageing and, at the same time, a condition of critical reflection on the existential advantages of longevity (Biggs & Haapala, 2016).

Summing up this brief review, we take the liberty to reason about cultural mitigation of loneliness in gerontological cohorts and the shift from a dominant negative, psycho-social mindset towards an existential one, interpreted as a resource for an ageing person’s self-realization.

This work is an attempt at considering the ageing person’s solitudes a multidimensional phenomenon, including analysis of the ability to find resources in situations of solitude, as well as the person’s attitudes to positive solitude as an existential fact.

## Conclusions

The investigation shows that experiencing solitude in the gerontological cohort is non-homogeneous, displays group specificity, and that the interconnection of personality attitudes to positive solitude with psychological well-being can lead to changes in the person’s activity and the extent of their experience of loneliness.

Regardless of their level of psychological well-being, the respondents displayed a trend towards accepting solitude, from the standpoint of actualizing its positive resources as an existential fact. At the same time, non-reflexive understanding of inactivity in old age from the standpoint of positive solitude, not supported by the “existential advantages of longevity”, may lead a person to a senseless position of ordinary existence or stereotypical copying of someone else’s life.

## Limitations and Prospects

Analyzing the results of this work critically, we find it interesting to consider them in the context of research limitations.

First, it is rather difficult to motivate an ageing person for voluntary “study of his/her life journey”, since many of them do not admit the uniqueness of that life journey and/or have strong doubts that their experiences are significant and valuable for society. By using only volunteers as subjects, with their degree of activity and motivation preserved, there is a risk of distortion of the research focus, as data concerning a less active gerontological group are lost.

Second, the open questions are not free of limitations such as inaccuracy and social desirability. This prevents us from getting a sufficiently comprehensive overview of the senior adults’ loneliness (solitude) in their everyday lives.

Third, there is a lack of methods adapted for old age. We consider this as a prospect for future research.

In conclusion, it should be noted that the research presented here has theoretical foundations and may provide an urgent field for further study of the positive aspects of solitude in old age, as a resource for resolution of existential and spiritual problems of this cohort.
